# Development of a New Control System for a Rehabilitation Robot Using Electrical Impedance Tomography and Artificial Intelligence

**DOI:** 10.3390/biomimetics8050420

**Published:** 2023-09-11

**Authors:** Alireza Abbasimoshaei, Adithya Kumar Chinnakkonda Ravi, Thorsten Alexander Kern

**Affiliations:** Institute for Mechatronics in Mechanics, Hamburg University of Technology, Eissendorferstr. 38, 21073 Hamburg, Germany; adithyakumarcr@hotmail.com (A.K.C.R.); t.a.kern@tuhh.de (T.A.K.)

**Keywords:** impedance tomography, home rehabilitation, control, artificial intelligence

## Abstract

In this study, we present a tomography-based control system for a rehabilitation robot using a novel approach to assess advancement and a dynamic model of the system. In this model, the torque generated by the robot and the impedance of the patient’s hand are used to determine each step of the rehabilitation. In the proposed control architecture, a regression model is developed and implemented based on the extraction of tomography signals to estimate the muscles state. During the rehabilitation session, the torque applied by the patient is adjusted according to this estimation. The first step of this protocol is to calculate the subject-specific parameters. These include the axis offset, inertia parameters, passive damping and stiffness. The second step involves identifying the other elements of the model, such as the torque resulting from interaction. In this case, the robot will calculate the torque generated by the patient. The developed robot-based solution and the suggested protocol were tested on different participants and showed promising results. First, the prediction of the impedance–position relationship was evaluated, and the prediction was below 2% error. Then, different participants with different impedances were tested, and the results showed that the control system controlled the force and position for each participant individually.

## 1. Introduction

According to the World Stroke Organization (WSO), stroke remains a major global health issue, ranking as the second most common cause of mortality and the third most frequent cause of both death and disability globally [1]. The impact of strokes is not only felt in terms of deaths, but also represents a significant economic burden, with an estimated global cost of more than EUR 721 billion [2]. The aftermath of a stroke can result in several limitations, including movement and speech problems, and changes in cognitive and emotional states. With the growing prevalence of conditions, such as stroke and spinal cord injuries, there is an increasing need for innovative rehabilitation techniques that can help patients recover their mobility and functionality [3], particularly relevant for patients undergoing extended training periods [4].

Advanced technology has greatly impacted the development of automated medical and rehabilitation systems [5,6]. This technology today allows patients to receive therapy and exercise programs remotely, which can be especially helpful for those who live in rural or remote areas, have mobility issues, or have limited access to physical therapy facilities. On the other hand, there is an increasing need for occupational therapists to assist patients with a wide range of exercises, so these devices can be used as remote rehabilitation systems to reduce the therapists’ workload [7]. Research also shows that remote rehabilitation can have several potential advantages, such as keeping patients engaged beyond the clinical context and tracking and assessing movements when they are at home [8]. Moreover, remote rehabilitation has the potential to reduce healthcare costs [7] by eliminating the need for patients to travel to physical therapy appointments and by reducing the need for in-person therapy sessions. Their design can also be optimized by combining parallel and serial mechanisms, or by using new methods to change the shape of the end effectors [9,10]. Therefore, remote robotic rehabilitation represents a promising area of research to improve the delivery and accessibility of physical therapy services [11].

Therapists must possess a deep understanding of the forces that affect repaired tendons and the tensile strengths of various repairs. Measuring the amount of flexion through conventional methods can be challenging. However, a rehabilitation device can gradually increase the amount of flexion, making it an ideal method for rehabilitation compared to traditional physical therapy [12,13,14]. These systems can be programmed to provide targeted exercises that help patients build strength, support compensation [15], improve range of motion and regain motor control. They have been shown to be effective in improving mobility, reducing pain, and enhancing the overall quality of life for patients undergoing rehabilitation [7,16,17,18]. In addition to these benefits, for robotic devices to perform better, they should be able to monitor the patient’s condition and provide valid data to the therapist so that he or she can get an idea of the patient’s progress.

Electrical impedance tomography (EIT) is a method that is based on the measurement of the electrical impedance of body tissues and can provide images of the internal structures of the body without using harmful radiation or invasive procedures [19]. EIT has been used in a variety of medical applications, such as monitoring lung function [20], detecting breast cancer [21,22] and monitoring brain activity [23]. However, it is not commonly used in robotic rehabilitation. The use of EIT technology in rehabilitation robotics has the potential to change the way medicine approaches rehabilitation, providing clinicians with a powerful tool for monitoring patient progress and tailoring rehabilitation programs to individual needs.

Given the various advantages of using remote robotic rehabilitation, this study presents a novel approach to design and produce a rehabilitation robot that restores wrist and forearm functionality through an automated rehabilitation protocol. In addition, the rehabilitation protocol proposed in this study uses EIT technology to evaluate muscle growth and an intelligent algorithm to update the patient’s hand model according to the patient’s improvement. The control system includes a mathematical model that describes the dynamics of forearm and wrist rotations [24] in three degrees of freedom and evaluates the torques of the forearm and wrist. The process of creating a model of the forearm and wrist enables evaluation of the rehabilitation progress and identification of the basic elements of the dynamic model. Therefore, the following three phases consist of these objectives: in the first phase, the system is calibrated with the patient’s healthy hand; in the second phase, the subject’s constant parameters are determined; and in the third phase, the patient is evaluated and exercises are performed. In the third phase, patients are asked to perform the desired movements, applying the necessary torque to move the robot in the desired direction. During therapy, the range of motion of the end effector and the electrical impedance of the forearm or wrist are measured. If the previous values are reached, the values are extended, and the new maximum values are increased for the next cycle. Otherwise, the current maximum values become the new endpoints. In the following, this paper will dive deeper into the details of the protocol by first explaining the design of the rehabilitation robot and then the model and control system, followed by the implementation of the tomography and finally the description of the protocol of the system and tests.

## 2. Methodology and Design

### 2.1. Mechanical Design

**Figure 1:**
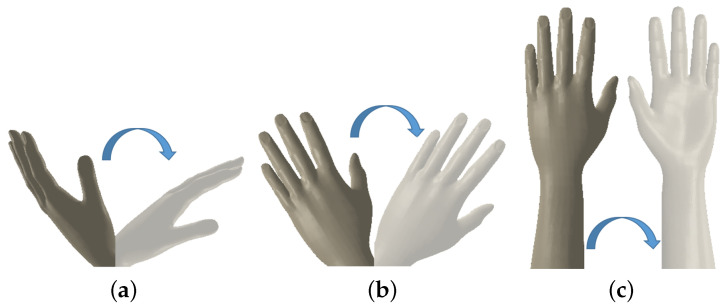
(**a**) Flexion/extension wrist movement. (**b**) Ulnar/radial wrist movement. (**c**) Supination/pronation forearm movement.

**Figure 2:**
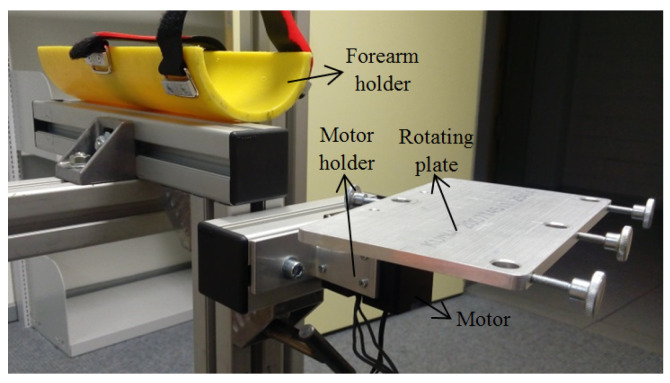
General view of the system.

The mechanical structure was designed with consideration of the anatomical constraints of forearm and wrist movements shown in Figure 1. Figure 2 shows the robot with the main components of the assembly listed in Table 1. Also, the system is adjustable in the X and Y directions to be useful for both hands. The design also took into account physical limitations to create a simple system with low weight.

**Figure 3:**
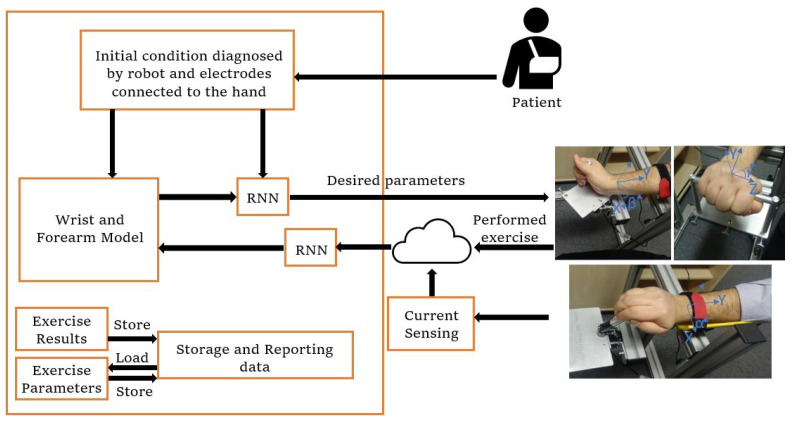
Control architecture design.

The model consists of six main parts. Each part with its elementary components is described in Table 1. The rotating plate is connected to the motor, which enables the movement of the hand. Unlike similar solutions, the robot is driven by a single motor that performs the three degrees of freedom (DOFs) of the forearm and wrist. We offer a new solution with 3 DOFs, using a single motor instead of three motors to drive the joint. Based on an interchangeable end effector, the robot enables the three basic movements of the wrist and forearm: flexion/extension, ulnar/radial, and supination/pronation. In fact, the distance between the forearm holder and the motor place can be adjusted in the horizontal and vertical directions. The actual state shown in Figure 3 and Figure S1 in the supplementary file provides different degrees of freedom by changing the end effector.

It shows the mechanical transformation that ensures the three basic wrist and forearm movements. The designed robotic system is user-friendly and straightforward to operate, requiring no specialized training for setup. It is a portable device that is simple to handle. Additionally, the robot is powered by a medium-torque Dynamixel motor, which supplies the necessary torque for implementing the innovative rehabilitation approach.

### 2.2. Mechanical Model of the Forearm and Wrist Rotations

This section introduces a mathematical model that captures the rotational dynamics of the forearm and wrist across three degrees of freedom (DOFs). It assesses the torques needed for these rotations and identifies key terms and parameters in the model that are essential for exercise applications. The model’s validity will be confirmed through experimental observations of forearm and wrist movements, conducted with a rehabilitation robot, to define the dynamics of these rotations.

The joints studied can be kinematically described as a universal joint with nonoverlapping axes (Figure S2 in the supplementary file). Supination–pronation occurs about the Y axis and is represented by α (Figure 3 and Figure S2 in the supplementary file). Flexion and extension is represented by β and occurs about the Z axis. Ulnar–radial deviation is represented by γ and occurs about the rotated X axis. As rotations occur, a series of torques are applied to the hand. Contraction of the muscles produces active torques. They are represented here as a group and resolved (without loss of generality) along the axes of supination/pronation (SP), flexion/extension (FE), and ulnar/radial (UR) (Figure S2 in Supplementary file). Passive torques result from the mechanical properties of the forearm and wrist, and gravity. The resulting torques are a combination of active and passive torques. To simplify the calculations, it is assumed that the damping and stiffness torques are linear and symmetrical about the neutral position.

Inverse kinematics is used for joint angles, where *SP*, *FE*, and *UR* represent the Euler angles of the forearm and wrist in supination/pronation, flexion/extension, and ulnar/radial movements, respectively. They are considered positive for pronation, flexion, and ulnar deviation. The frame XYZ is fixed in the hand (third metacarpal joint) and rotates with the hand. This results in the following equations of motion, which relate the torque in each DOF to the resulting motion:

(1) $$\begin{array}{c} \left[\begin{array}{c} \tau_{SP}\\ \tau_{FE}\\ \tau_{UR} \end{array}\right]=\left[\begin{array}{c} \frac{\partial \tau_{SP}}{\partial \theta_{SP}}\theta_{SP}+\frac{\partial \tau_{SP}}{\partial \theta_{FE}}\theta_{FE}+\frac{\partial \tau_{SP}}{\partial \theta_{UR}}\theta_{UR}\\ \frac{\partial \tau_{FE}}{\partial \theta_{SP}}\theta_{SP}+\frac{\partial \tau_{FE}}{\partial \theta_{FE}}\theta_{FE}+\frac{\partial \tau_{FE}}{\partial \theta_{UR}}\theta_{UR}\\ \frac{\partial \tau_{UR}}{\partial \theta_{SP}}\theta_{SP}+\frac{\partial \tau_{UR}}{\partial \theta_{FE}}\theta_{FE}+\frac{\partial \tau_{UR}}{\partial \theta_{UR}}\theta_{UR} \end{array}\right]=\\ \left[\begin{array}{ccc} \frac{\partial \tau_{SP}}{\partial \theta_{SP}} & \frac{\partial \tau_{SP}}{\partial \theta_{FE}} & \frac{\partial \tau_{SP}}{\partial \theta_{UR}}\\ \frac{\partial \tau_{FE}}{\partial \theta_{SP}} & \frac{\partial \tau_{FE}}{\partial \theta_{FE}} & \frac{\partial \tau_{FE}}{\partial \theta_{UR}}\\ \frac{\partial \tau_{UR}}{\partial \theta_{SP}} & \frac{\partial \tau_{UR}}{\partial \theta_{FE}} & \frac{\partial \tau_{UR}}{\partial \theta_{UR}} \end{array}\right]\left[\begin{array}{c} \theta_{SP}\\ \theta_{FE}\\ \theta_{UR} \end{array}\right] \end{array}$$

Then, the following formula and table are obtained:

(2) $$\begin{array}{c} \left[\begin{array}{c} \tau_{SP}\\ \tau_{FE}\\ \tau_{UR} \end{array}\right]=\left[\begin{array}{c} A \end{array}\right]\left[\begin{array}{c} \ddot{\alpha }\\ \ddot{\beta }\\ \ddot{\gamma } \end{array}\right]+\left[\begin{array}{c} B \end{array}\right]\left[\begin{array}{c} \dot{\alpha }\\ \dot{\beta }\\ \dot{\gamma } \end{array}\right]+\left[\begin{array}{c} C \end{array}\right]\left[\begin{array}{c} \alpha \\ \beta \\ \gamma \end{array}\right]+\left[\begin{array}{c} D \end{array}\right] \end{array}$$

In Equation (2), the left-hand side signifies the overall active torques, attributable to muscle contractions. These torques could be influenced by both displacement and its rate of change, as well as neuronal activation. This is because neuronal activation has an impact on muscle stiffness and damping characteristics. [*A*] shows the inertial torque, [*B*] is the damping torque, [*C*] represents the stiffness torque, and [*D*] is for gravitational torques and those associated with coupled rotations. Since the system is an intelligent system, it can adapt to the hand situation after a few trials. Therefore, we used the average values for men and women for the coefficients [25]. Thus, the matrix values are found and given in the equations in the supplementary file.

For the numbers, we used the averaged values. As an example, based on the related literature [26], the flexion axis was measured 3.9 ± 2.0 mm, and 3.9 ± 1.4 mm was measured for the extension axis of male. In this work, all male subjects were given 4 mm.

### 2.3. Control Architecture

As shown in Figure 3, the main architecture contains different subsystems. The flow begins with data received from the patient and diagnosis of the initial condition by electrodes connected to the hand. Based on the electrode data, the learning algorithm sets a target for the current exercise. In parallel, the wrist and forearm model sends the initial torque signal to the neural network to define the initial parameters and send the signal to rotate the motor. According to the performance of the patient and the sensor signals, the model parameters are updated in real time during the experiment with the recurrent neural network algorithm. Based on the new model, the error between the target and the current position, and the torque input from the patient, the new signals are sent from the recurrent neural network to the robot and define the force that should be exerted on the hand, and this loop continues to reach the target.

The model of the hand is dynamically refreshed to compute the resulting torque for the entire system. This resulting torque is determined by taking the estimated muscle-applied torque and subtracting the output torque predicted by the model. Thus, the initial and feedback values affect the updated forearm and wrist model each time. According to the model parameters, an output torque is estimated and from the subtraction of this torque by the torque applied by the muscle, the resultant torque is determined. The flow chart for this can be found in Figure S3 in the supplementary file.

**Figure 4:**
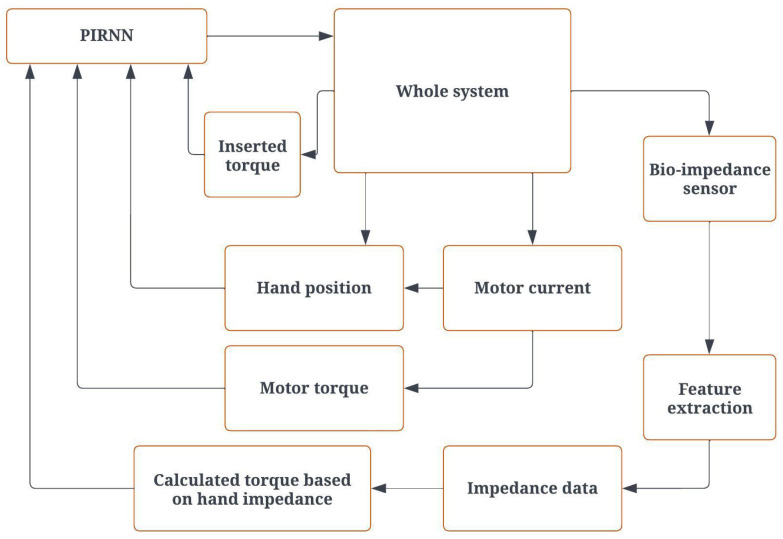
Robot control architecture.

**Figure 5:**
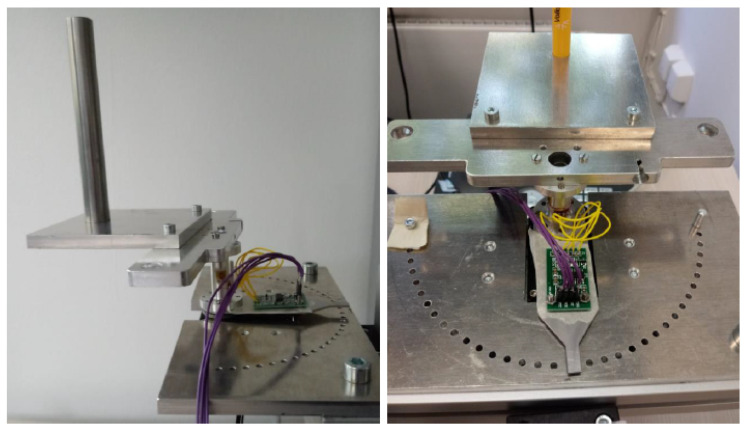
Sensor assembly from side and back views.

On the other hand, the necessary motor torque is derived from the latest sensor impedance readings and the current hand position, as captured by the encoder linked to the Dynamixel motor. As depicted in Figure 4, the recurrent neural network (RNN) learning algorithm evaluates various inputs, such as the present joint position, applied torque, motor-generated torque, and torque assessed based on hand impedance. It then transmits the updated target values to the robot. For the purpose of measuring the patient-applied torque, a strain gauge setup is designed and put into action, as shown in Figure 5.

Incorporated into this system is an adaptive learning algorithm, specifically a recurrent neural network (RNN), which plays a pivotal role in real-time adaptability. This algorithm processes multiple forms of input data. Based on these inputs, the RNN continually updates the control signals and model parameters, thereby tailoring the rehabilitation process to each individual patient’s needs and performance metrics. Such real-time adaptability not only enhances the precision of the rehabilitation process but also contributes to the system’s overall robustness.

Regarding safety concerns, a multi-faceted approach is implemented to ensure that both the mechanical and software aspects of the system operate within safe parameters. On the mechanical front, mechanical stops are integrated into the system that act as hard limits to prevent any unintended or extreme movements by the robot, thereby enhancing physical safety.

From an electrical standpoint, the system continuously measures the current flow during operation. If the current exceeds a predetermined safe threshold, the system automatically stops all movements, serving as an electrical safety measure.

On the software side, some algorithms are designed that monitor these safety parameters in real-time and take corrective action as needed. Moreover, an emergency stop button is incorporated into the system interface. This allows the users to immediately stop all operations if they feel discomfort or perceive any form of risk, providing an additional layer of user-controlled safety.

These safety measures collectively provide a robust framework that prioritizes the well-being of the user while ensuring that the system operates within safe and effective boundaries.

### 2.4. Tomography

Tomography is a method of scanning cross sections by penetrating waves. It is used to evaluate the situation of the user’s wrist and forearm. It calculates the resistance between two poles of an electrode, and the result of this measurement is called bio-impedance. The sensor chosen for impedance measurement is the AD5933 impedance converter and network analyzer, also called an impedance sensor. This sensor was chosen because of its communication protocol, which sends data directly to the Arduino via an I2C bus connection. The sensor is inexpensive and reliable. Thanks to its small size of 4.1 cm × 2.0 cm, we can easily mount it anywhere. As part of the data input for the analysis, a PmodIA impedance analyzer with an integrated AD5933 is used to measure the impedance between two points [27].

#### 2.4.1. Impedance Analyzer Setup

**Figure 6:**
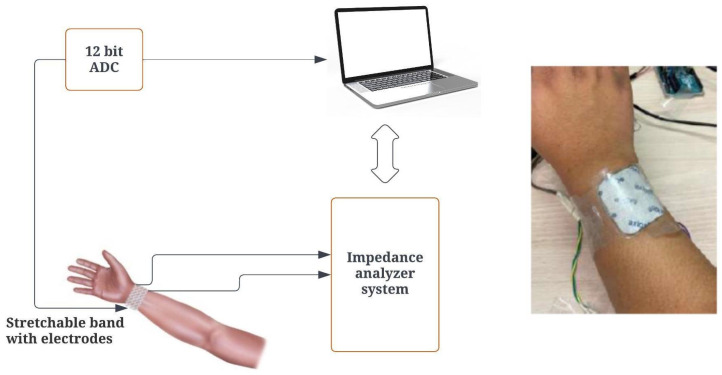
Impedance analyzer system setup.

An AD5933 impedance analyzer is a 12-bit analyzer with a measurement range of 100 Ω to 10 MΩ. The PmodIA employs the AD5933 to stimulate an unidentified external impedance at a predetermined frequency, utilizing its integrated frequency generator and analog-to-digital converter (ADC). The frequency is fed internally via one of the subminiature version A (SMA) connectors. Other SMA connectors capture the frequency response and transmit it to the ADC. The sampled data undergo a discrete Fourier transform (DFT), after which the real and imaginary components of the outcome are processed within the on-chip data registers [27,28]. Using I2C interfaces, we can communicate with a microcontroller, such as Arduino, and extract data. In this case, we used the Arduino Uno. The choice of the Arduino Uno for this project is due to its form factor and lower cost. The PmodIA acts as the slave device, and the Arduino acts as the primary device that communicates using the I2C protocol. Figure S4 in the supplementary file shows the schematic and implemented connection between PmodIA and the Arduino microcontroller, and Figure 6 shows the concept and setup on hand.

#### 2.4.2. Impedance Calculations

The analog-to-digital converter starts a frequency sweep at each point to calculate the unknown frequency response at up to 100 kHz with a resolution of 12 bits. Before the measurements are evaluated, a DFT is applied to the sampled data at each frequency step. Two values are stored in each register, one real and one imaginary. The electrical impedance can be expressed with Equation (3):

(3) $$\begin{array}{c} Z=Real+j*imaginary \end{array}$$

The first step in impedance is to calculate the amount, which can be calculated using Equation (4):

(4) $$\begin{array}{c} Magnitude=\sqrt{\left(R^{2}+I^{2}\right)} \end{array}$$

*R* is the real number stored at register addresses 0 × 94 and 0 × 95. *I* is the imaginary number stored at register addresses 0 × 96 and 0 × 97. The built-in ADC and DFT capture the response signal, which is then processed by a digital signal processor. At each output frequency, the DFT function returns real and imaginary data. Impedance is calculated by inverting the product of the gain factor and magnitude. Note that the gain factor is calculated during the calibration process of the system, where a known impedance is connected:

(5) $$\begin{array}{c} Impedance=\frac{1}{GainFactor*Magnitude} \end{array}$$

Since the sensor has a finite series of frequency responses, the gain factor may vary with frequency. This can lead to an error in a certain frequency range. To reduce the error, we keep the frequency range as low as possible, and a low frequency is sufficient for our goal of estimating the hand impedance.

#### 2.4.3. Torque Calculation Based on Hand Impedance

Based on the estimated active stiffness and impedance of the hand, the torque provided by the end effector should be adjusted:

(6) $$\begin{array}{c} T=C\omega +KE \end{array}$$

where *C* is the damping coefficient, ω is the angular velocity, *K* is the stiffness, and *E* is the error between the target position and the actual position. The torque (*T*) is determined by this formula and helps the patient to reach the target point. It helps in providing adaptive and personalized therapy for all individuals, as the degree of disability and physical abilities are inherently different. By telling the patient the level of assistance, they are motivated to keep better records. The controller should learn this in real time and provide an appropriate assistance torque to accomplish the task. Therefore, the robot should provide higher assistance and lower compliance when rehabilitating severely impaired patients, and high compliance and lower assistance as the patient improves.

**Figure 7:**
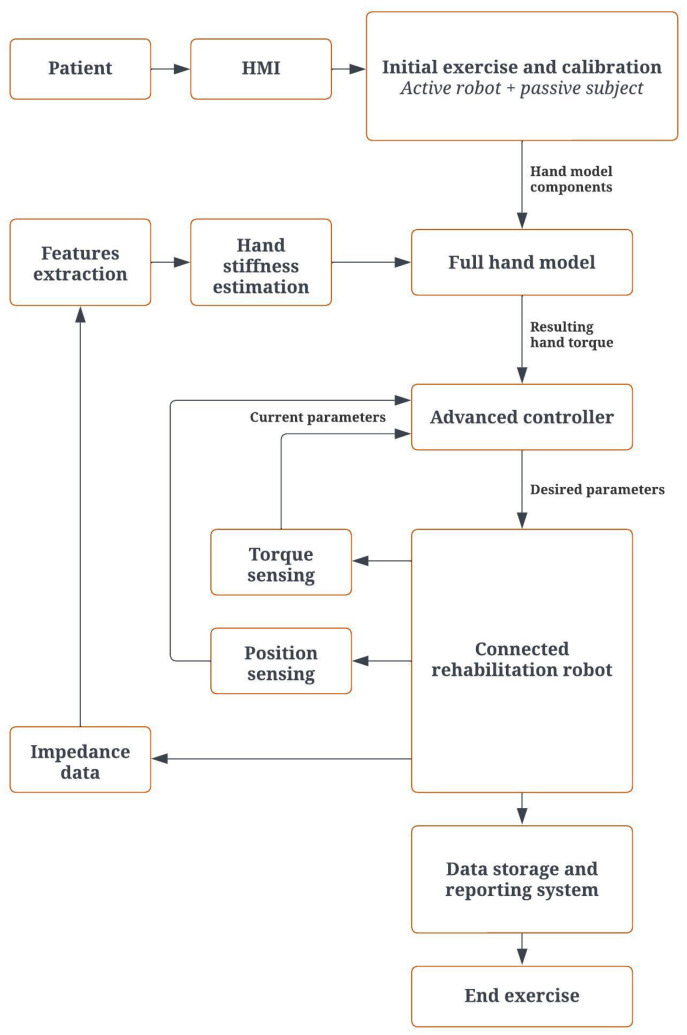
Operating system flow chart.

The patient starts the control process by entering the desired settings. After appropriate processing, the required parameters are transmitted to the advanced control block implemented in the human–machine interface (HMI). The robot receives the calculated parameters, including the required torque and position. The board processes the data from the sensors and encoder before sending them back to the controller. Then, the extracted impedance features are used to estimate muscle stiffness. The motion control block uses position feedback to make the correct choice. The settings and results are stored in the database. Figure 7 shows the operational flowchart of the system. The robot manages the torque and data in the designed architecture. In this case, the calculated torque is the topic to be published.

#### 2.4.4. Protocol Description

The first step is the calibration of the system using the patient’s electrical impedance. The subject must use the healthy arm to calibrate the impedance values. During the second step, the impedance values are used by the system to calculate the subject’s constant parameters for the wrist and forearm model. The third step is to perform exercises and evaluate the patient’s condition and rehabilitation progress. The subject must perform the desired exercises and apply the necessary torque to move the robot to the desired point. In this case, the robot measures the range of motion of the end effector and the electrical impedance of the lower arm and compares them with the previous values. If the subject cannot achieve the previous values, the current maximum values are set as endpoints. If the previous values are achieved, the desired values are extended, and the new maximum values are increased for the next cycle.

## 3. Results and Discussion

The designed robot is equipped with only one motor, cost-effective, practical, and easy to use, which makes it an ideal option for in-home rehabilitation. The fact that it is automatically controlled makes it easier for the users to use it whenever they want. The system security is enhanced by this personalized control system. Based on the passive training and impedance data, the system develops the hand model, and the control flow is started.

Before starting, the patient should select a path to save the report file and enter the name and age. Note that these parameters are automatically stored in the corresponding database and will be imported later in the next exercises. The patient can update these parameters at any time.

The next step is to calibrate and drive the hand model by starting the first exercise. The system uses one-shot learning and must be calibrated for each person. Impedance values may vary depending on the muscle situation and physical characteristics of the user. From experimental observations, they change every time and do not always remain the same. Therefore, it is advisable to calibrate the system each time before starting the training. Each time the software is started, the calibration process must be repeated. This is to ensure that the system remains calibrated at the beginning of each session and provides more safety.

After finalizing the component measurements, the next thing is to start the exercise. By clicking on a button, the software starts the exercise. First, the software checks if there are values for the angle range. Using the impedance prediction and the current impedance data, it also checks if it is possible to apply less assistance force to the end effector. When patients can no longer move their hands, the system stops and sets that as the endpoint for the day. The same is performed for both sides from the center for each DOF. For existing patients, the values are compared with the previous results to look for improvements. For each additional trial, the system increases the values and checks if the user can move, just as the therapist does in the clinic. If the user is comfortable with these new values, it updates the sheet. The range of motion achieved and the assistance torque generated are stored to evaluate the degree of rehabilitation. In case of an emergency, the program can be stopped at any time by pressing the emergency stop button. This is an additional safety option for the user. In addition, for each DOF, the maximum angle is defined, beyond which the system cannot go. The ranges for flexion, extension, ulnar and radial deviation, pronation, and supination are defined as 85°, 80°, 30°, 20°, 90°, and 90°, respectively, and can be changed in the code.

The report is self-generated. It provides daily records of the user’s progress, giving physicians an overview of the improvement in the user’s health. As shown in Table 2 (name and age are removed because of privacy), it helps the physician to understand how the patient has been coping with the exercises of the intelligent wrist therapy robot. It is also consulted by the software to track the user’s previous limitations. The report is generated for each new user, and if the user has already used the system, the system adds each day’s limits.

Because this is the user’s private data, this system places the highest value on privacy and informed consent. It uses Google Cloud Platform for data storage and transfer, which follows strict security protocols. For secure data transfer between the device and the cloud, we use SSL/TLS encryption methods to ensure that patient data remain confidential and protected from unauthorized access. In addition, we use Google Cloud’s Identity and Access Management (IAM) features to control who has access to different types of data and to ensure that only authorized personnel can enter the application and perform tests.

To maintain ethical standards, our system also requires user consent at every crucial step where data are collected or transferred. This ensures that users know exactly how their data will be used, stored and protected, in line with best practice for informed consent.

**Figure 8:**
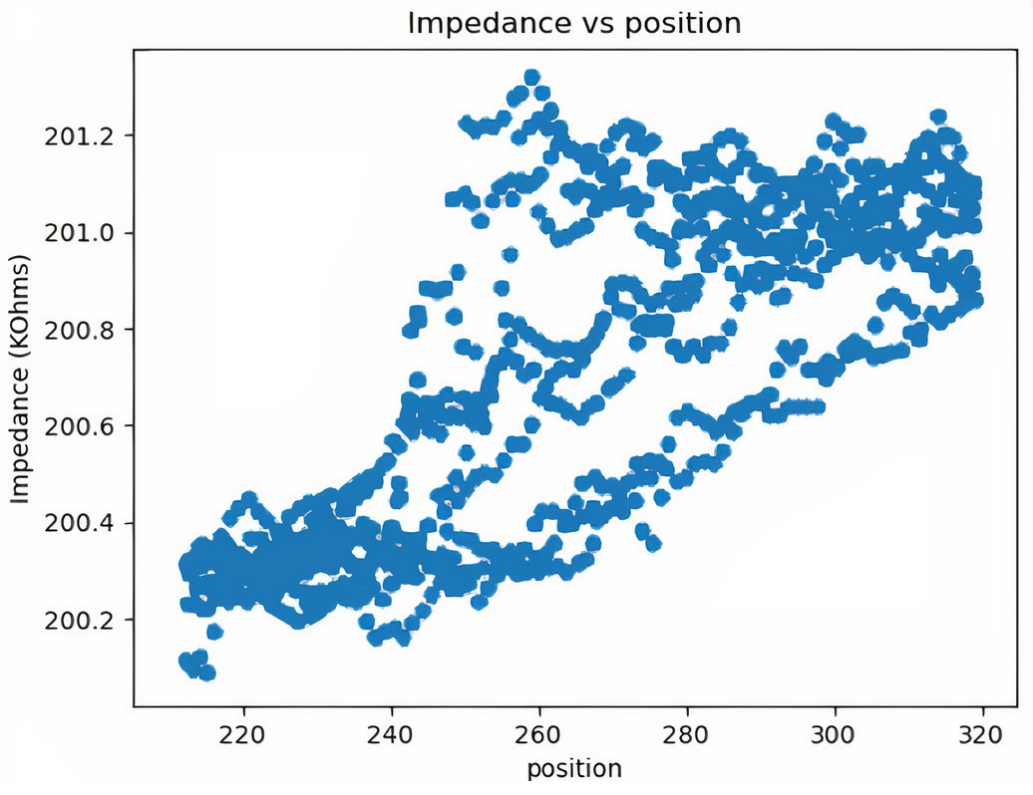
Impedance variation across different positions before noise filtering.

To predict the impedance–position relationship of the hand, a direct comparison between position and impedance is made, and a pattern between impedance and position is observed. The behavioral diagram is shown in Figure 8.

**Figure 9:**
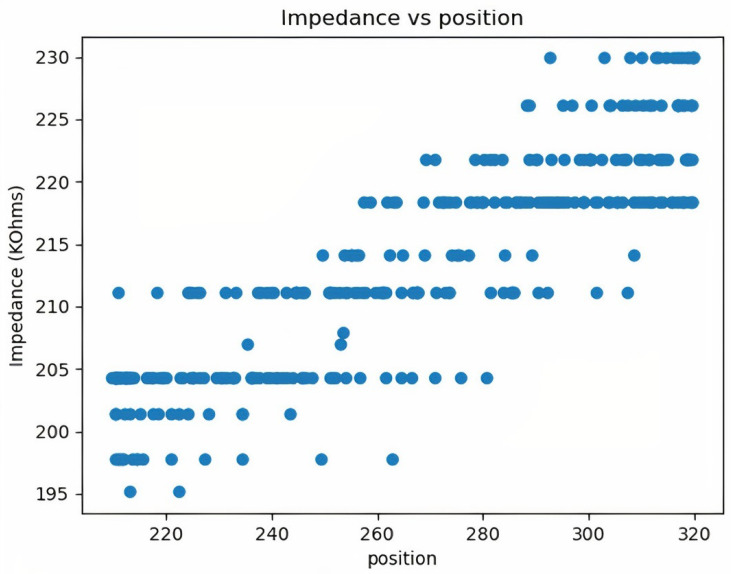
Impedance variation across different positions after noise filtering.

The pattern between impedance and position shown in Figure 8 is the raw data available directly from the Arduino microcontroller. These values are generally clustered and contain high sensor errors. To achieve higher accuracy and better measurements, slope-based filtering is used to eliminate noise. As can be seen in Figure 9, there is a pattern between position and impedance. This pattern is a relationship between a mechanical quantity (position) and an electrical quantity (impedance). Since the two quantities come from two different areas, determining the relationship between them is complex. Using machine learning algorithms and a large data set, it is possible to establish a relationship and predict the impedance value based on healthy human behavior. Since the process of generating relationships is automated, the whole process can be easily reused for other purposes, e.g., to add additional exercises. This is helpful for the future development of the system.

**Figure 10:**
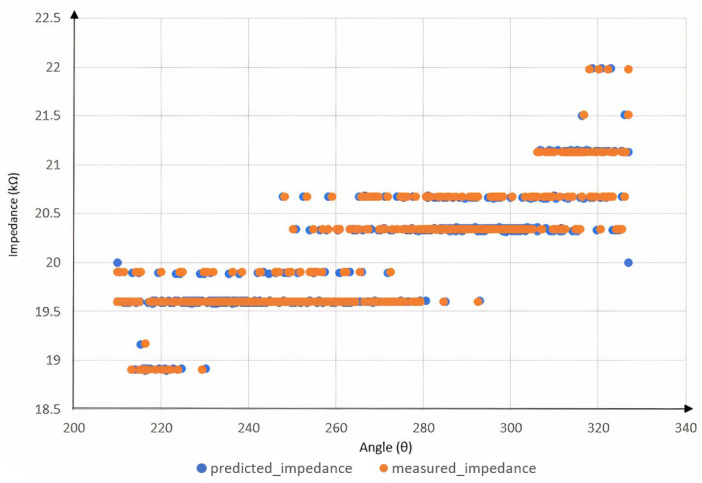
Comparison of predicted impedance and impedance with respect to angle.

Running the model with different degrees of polynomials, we find that using a fifth-degree polynomial minimizes the mean average error. The first experiment is to check the accuracy of the model by comparing the predicted and actual impedance. Note that the value of the impedance depends on the position of the electrodes, the temperature, the time of day and the condition of the body. After various trial and error methods, it is found that the electrodes must be placed about 6.5 cm below the wrist. Both electrodes must face the opposite side. In addition, the fit should be neither very tight nor very loose, and it should be comfortable while making firm contact with the skin. Since other conditions, such as temperature and body condition, cannot be controlled, it is advisable to fit the curve with the initial impedance values. The first 20 values are compared so that the actual impedance and the predicted impedance are on the same line. The time needed for this process is only a fraction of a second. Therefore, it does not interfere with our work. Figure 10 shows the measured and predicted impedance. As can be seen from the results, the predicted points are very close to the measured points. The test is performed several times at different times of the day and week to ensure the stability of the system. No change in prediction accuracy is observed, and the predictions give good results with an error of less than 2%.

The next test is to ensure that the system is able to shut down if the user applies too much force to the system. If the patient is in pain and cannot move his hand, it can be seen that the difference between the predicted and measured impedance slowly grows.

**Figure 11:**
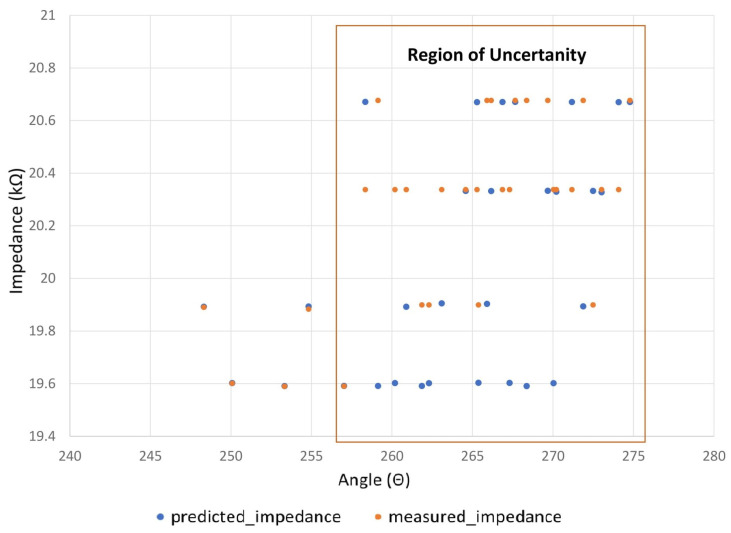
Region of uncertainty.

In such cases, the system is brought to a standstill. The program written for this research continuously monitors both the predicted and the actual impedance. If the impedance increases or if the impedance values differ from the predicted impedance, the system stops the entire process. To test the concept, an experiment is conducted to check the performance of the system where the degree of wrist movement is limited. Figure 11 illustrates this situation. When the movement is restricted after 260, there is a slow deviation between the predicted impedance and the impedance. The impedance value should be lower for a normal person. However, due to simulated uncertainty, a certain counterforce that restricts the movement of the motor causes the impedance value to increase. Once the system exceeds the limit, it shuts down to stop this process. This experiment is carried out several times to check the reliability of the system.

**Figure 12:**
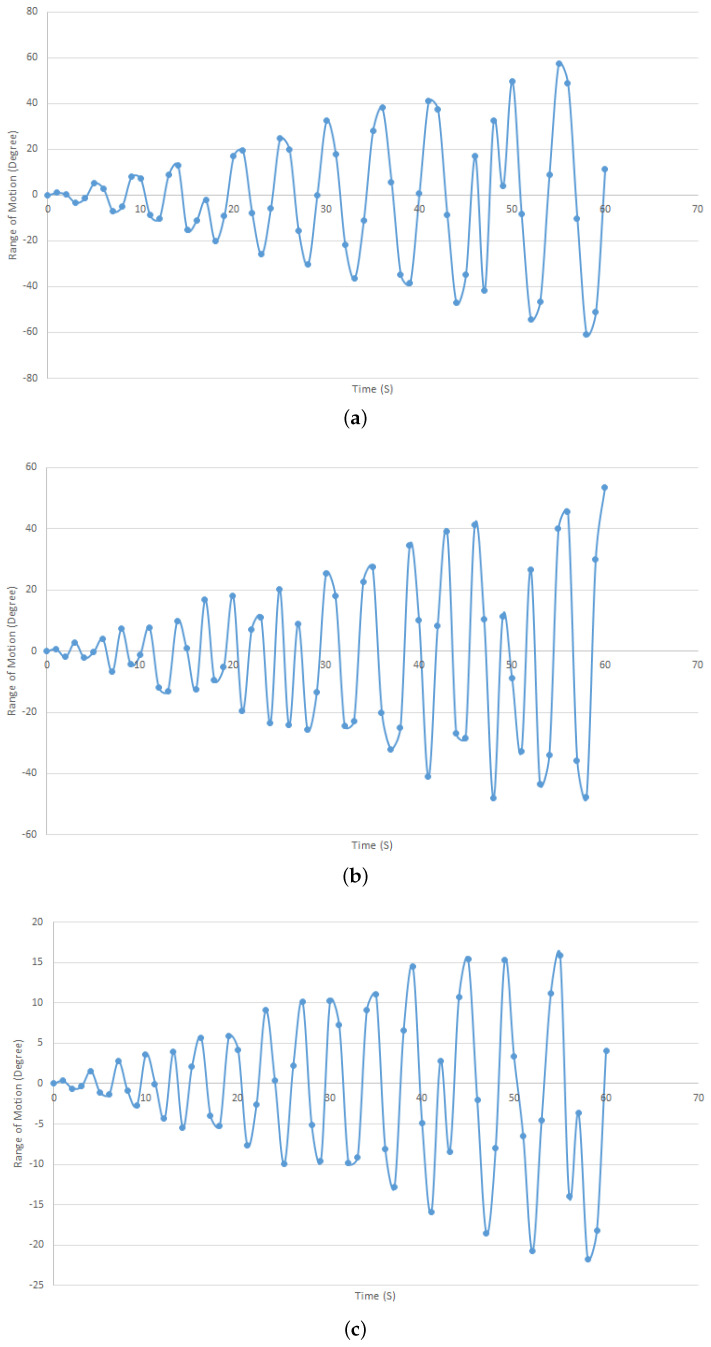
Navigating the participant hand to the desired position. (**a**) Lowest impedance. (**b**) Medium impedance. (**c**) Highest impedance.

In order to test the controller’s ability to control position in relation to impedance, three different participants were tested with three different impedances as part of the robot training described. The aim of this experiment was to see if the system could behave differently depending on the impedance. Figure 12a–c represent the lowest impedance, the medium impedance, and the highest impedance, respectively, and they show the results of the training in the first minute for comparison.

As can be seen from the data, the movement with the lowest impedance within one minute covers a larger range of motion than the others, and the user has more ability to move his hand than others. In addition, the change and reaching of the target is smooth so as not to harm the users, and therefore the position increases gradually. Although there are some cases where the system returns to a previous state during movement, these are rare and have evolved due to impedance detection. The robot has not moved beyond the predefined angular limits for each DOF.

In order to test the ability of the controller to control the force applied to the hand as a function of different impedances, we tested it with two different impedances. The assumption is that the control system with a higher impedance should exert more force on the hands, as this is exactly what physiotherapists do. We also set a threshold for stopping the force so as not to harm people. Comparing the results of the force–torque sensor between these two impedances shows that as the impedance increases, the force and torque also increase (see the supplementary file for more details and the corresponding Figures S5 and S6 ).

**Figure 13:**
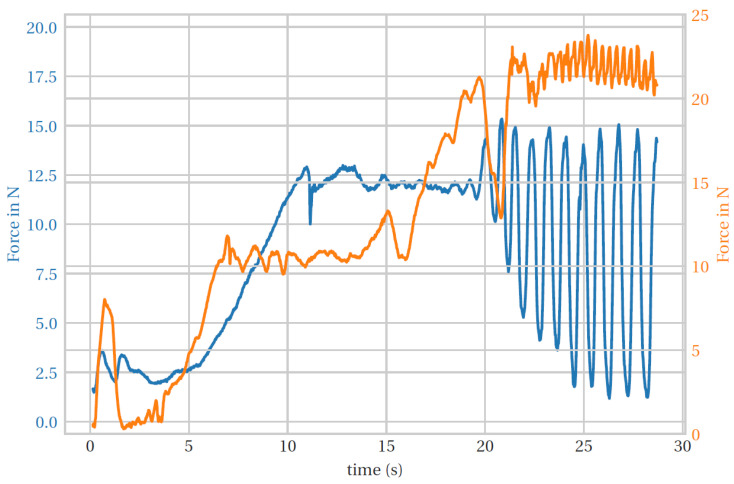
Average force comparison (blue is higher impedance and orange is lower impedance).

We carried out various tests with two different impedances. As can be seen in Figure 13 as an average of the results, the forces increase with increasing impedance, e.g., at 7.5 s the force is 6.558 N at low impedance and 10.454 N at higher impedance, almost 4 N more and 37% more. Also, at 20 s, the force is 13.59 N at lower impedance and 19.16 N at higher impedance, almost 6 N more, an increase of 29%. On average, the force is increased by 33% when the impedance is doubled, which shows that the controller controlled the force well after detecting the impedance.

Traditional rehabilitation methods come with inherent limitations, such as the requirement for one-on-one sessions with healthcare providers and the absence of monitoring during unsupervised exercises. These drawbacks often compromise the effectiveness of the treatment and increase the risk of relapse. In contrast, our proposed robotic system aims to mitigate these issues by offering smart, adaptive guidance. This feature allows patients to engage in rehabilitation tasks independently, with the robot offering real-time feedback and making dynamic adjustments to the exercise parameters. Such adaptability ensures that patients are continuously challenged according to their individual progress.

To further enhance the system’s utility, a user-centered design approach was employed. We conducted a survey involving prospective end users to gather insights into their specific needs and preferences. These findings were integrated into the system’s design to make it not just easy to use but also more effective in meeting rehabilitation goals. This approach underscores our commitment to delivering a system that supersedes traditional methods not only in terms of efficiency but also in the quality of care provided.

Compared to similar controllers in the field of rehabilitation robotics, this system has some advantages. For example, there are some systems that use robust and adaptive control [29,30] which have made important contributions to the field. However, our approach is characterized by its adaptability and stability, which are mainly facilitated by the integration of artificial intelligence systems and self-correction mechanisms. Unlike traditional control architectures, our AI-driven model allows real-time adjustments based on complex tomography signals, providing a better understanding of muscle health and activity. This capability improves both the predictive accuracy and safety of the rehabilitation process. In addition, the system’s self-correction function adapts to the changing impedance of the patient’s hand, ensuring that the applied torque remains within a safe and effective range. Overall, these advances contribute to more personalized, robust and reliable rehabilitation.

## 4. Conclusions

Advancements in rehabilitation robotics have made significant progress in recent decades. Through the use of technical solutions, experts aim to facilitate the restoration of body functions. Given recent developments in artificial intelligence and tomography, it is necessary to combine these with rehabilitation. In this context, we propose here an automatic control system to control a rehabilitation robot for the wrist and forearm. A new protocol was proposed to determine the health status of the hand and the dynamic parameters of the human–robot interaction. Muscle stiffness during the course of rehabilitation treatment directly affects the torque produced by the patient. The suggested method acknowledges this constraint and incorporates a control mechanism that evaluates muscle stiffness levels and adjusts the estimated torque values in real time. To manage the robotic system and offer data storage and reporting capabilities, a human–machine interface was designed and implemented. Tests were conducted with different participants and showed encouraging results. The results indicate that the system can predict the relationship between impedance and position within an acceptable range and also control the position and force according to the user’s impedance.

One of the notable advantages of this system lies in its cost effectiveness. Utilizing a single motor to achieve three degrees of freedom not only simplifies the mechanical design but also significantly reduces the hardware costs. Furthermore, the system is optimized for home-based rehabilitation, eliminating the need for patients to make frequent, potentially costly visits to healthcare providers. This home-based approach also allows healthcare providers to manage multiple patients more efficiently, thereby reducing operational costs. The convenience of a home-based system additionally means that patients can engage in rehabilitation exercises at their own pace and on their own schedule, further adding to the system’s overall cost effectiveness and patient-centric design.

## 5. Future Work

The current study lays the foundation for a robust and adaptive control system for rehabilitation robots, but several avenues remain open for future research. One possible direction is to refine the regression model that estimates muscle state based on tomography signals, possibly incorporating other imaging techniques or biosensors to gain a more comprehensive understanding of muscle health and activity. Exploring the scalability of the system to other types of rehabilitation or even completely different medical applications could also be a promising way to expand the impact of this technology.

Although robustness and reliability were considered in the development of the system, it is important to identify potential real-world challenges that could affect its practical application. These include system maintenance requirements, technical issues and the need for regular software updates to meet changing rehabilitation needs. Furthermore, any healthcare technology is only truly tested through its actual application. Therefore, extensive usability testing with multiple patients and therapists is planned for future studies. This will provide invaluable insights into the usability and effectiveness of the system and will serve to further refine both the technical elements and the user interface.

## 6. Patents

Abbasimoshaei, Alireza, and Kern, Thorsten Alexander; “Rehabilitation Apparatus for wrist and forearm therapy”; International Publicational Number: WO 2022/048906 A1.

## Figures and Tables

**Table 1:** Main mechanical components.

Piece Name	Material	Quantity
Forearm holder	Plastic	1
Motor holder	Steel	2
Motor	Dynamixel motor	1
Rotating plate	Aluminium	1
Horizontal rail	Aluminium	1
Motor place regulator	Aluminium	1

**Table 2:** Patient exercise report.

Name	Age	Date	Time	Repetition	Flexion	Extension	Ulnar	Radial	Supination	Pronation
		27022023	09:47:00	10	10	5	5	3	20	15
		27022023	21:16:00	6	12	8	5	4	23	17
		28022023	08:56:00	8	13	8	7	5	27	19
		28022023	18:34:00	12	15	10	9	6	29	19
		29022023	10:12:00	3	16	12	12	8	32	21

## Data Availability

All data and code will be made available on request to the correspondent author’s email with appropriate justification.

## Supplementary Materials

The following supporting information can be downloaded at: https://www.mdpi.com/article/10.3390/biomimetics8050420/s1, Figure S1: A general view of the manufactured device in different movements; Figure S2: Diagram of the forearm and wrist model; Figure S3: Resulting torque estimation flowchart; Figure S4: Connection of sensor and Arduino; Figure S5: Corresponding forces and torque for lower impedance; Figure S6: Corresponding forces and torque for higher impedance.
