# Supplementary material for: Development of a New Control System for a Rehabilitation Robot Using Electrical Impedance Tomography and Artificial Intelligence
Source: Biomimetics (Basel). 2023 Sep 11;8(5):420. doi: 10.3390/biomimetics8050420 (PMC10526263; doi:10.3390/biomimetics8050420)
Supplement: Supplementary file 1 [file biomimetics-08-00420-s001.zip › biomimetics-2584988-supplementary.pdf]

biomimetics

MDPI

Article

Supplementary file for paper: Development of a New Control System for a Rehabilitation Robot Using Electrical Impedance Tomography and Artificial Intelligence

Alireza Abbasimoshaei*, **Adithya Kumar Chinnakkonda Ravi** and **Thorsten Alexander Kern**

Institute for Mechatronics in Mechanics, Hamburg University of Technology, Eissendorferstr. 38, 21073 Hamburg, Germany; adithyakumarcr@hotmail.com (A.K.C.R.); t.a.kern@tuhh.de (T.A.K.)
* Correspondance: al.abbasimoshaei@tuhh.de; Tel.: +49-40-428-78-3083.

Abstract: In this study, we present a tomography-based control system for a rehabilitation robot with a new protocol for determining progress and a dynamic model of the system. In this model, the torque generated by the robot and the impedance of the patient's hand are used to determine each step of the rehabilitation. In the proposed control architecture, a regression model is developed and implemented based on the extraction of tomography signals to estimate the muscles state. Based on this estimation, the patient's torque is updated during the rehabilitation session. The first step of this protocol is to calculate the subject-specific parameters. These include the axis offset, inertia parameters, passive stiffness and passive damping. The second step is to determine the remaining components of the model, including the interaction torque. In this case, the robot calculates the torque generated by the patient. The developed robot-based solution and the proposed protocol were tested on different participants and showed promising results. First, the prediction of the impedance–position relationship was evaluated, and the prediction was below 2% error. Then, different participants with different impedances were tested, and the results showed that the control system controlled the force and position for each participant individually.

Keywords: impedance tomography; home rehabilitation; control; artificial intelligence

1. Mechanical Model of the Forearm and Wrist Rotations

To get a better idea of the angles and rotations of the hand, we show the actual system and a schematic view of the system with the corresponding angles in Figures S1 and S2.

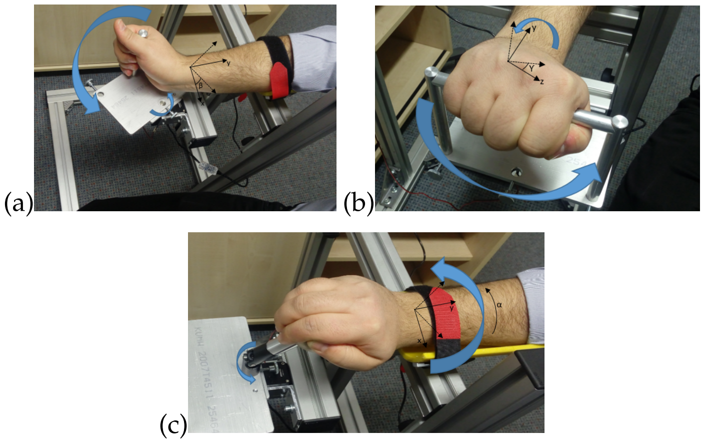

Figure S1. The manufactured device. (a) Flexion/extension wrist movement. (b) Ulnar/radial wrist movement. (c) Supination/pronation forearm movement.

As mentioned in the main text of the paper, joints are considered kinematically as universal joints. Figure S2 shows the simplified model of the hand and axes of supination/pronation (SP), flexion/extension (FE), and ulnar/radial (UR).

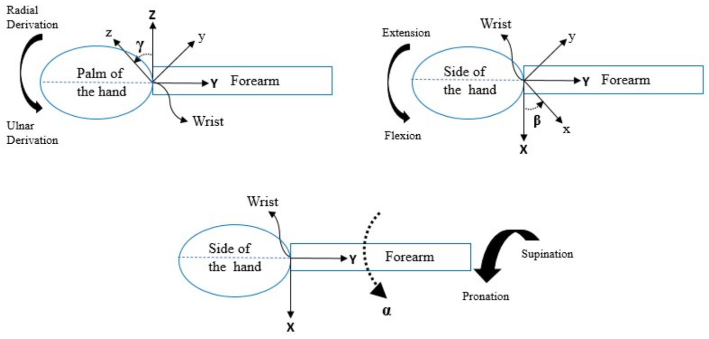

Figure S2. Diagram of the forearm and wrist model.

Inverse kinematics is applied to determine the joint angles. As a consequence, we obtain the subsequent equations of motion that establish the relationship between the torque in each degree of freedom and the resulting motion:

$$\begin{bmatrix}\tau_{SP}\\ \tau_{FE}\\ \tau_{UR}\end{bmatrix} = \begin{bmatrix}\frac{\partial\tau_{SP}}{\partial\theta_{SP}}\theta_{SP} + \frac{\partial\tau_{SP}}{\partial\theta_{FE}}\theta_{FE} + \frac{\partial\tau_{SP}}{\partial\theta_{UR}}\theta_{UR}\\ \frac{\partial\tau_{FE}}{\partial\theta_{SP}}\theta_{SP} + \frac{\partial\tau_{FE}}{\partial\theta_{FE}}\theta_{FE} + \frac{\partial\tau_{FE}}{\partial\theta_{UR}}\theta_{UR}\\ \frac{\partial\tau_{UR}}{\partial\theta_{SP}}\theta_{SP} + \frac{\partial\tau_{UR}}{\partial\theta_{FE}}\theta_{FE} + \frac{\partial\tau_{UR}}{\partial\theta_{UR}}\theta_{UR}\end{bmatrix} = \begin{bmatrix}\frac{\partial\tau_{SP}}{\partial\theta_{SP}} & \frac{\partial\tau_{SP}}{\partial\theta_{FE}} & \frac{\partial\tau_{SP}}{\partial\theta_{UR}}\\ \frac{\partial\tau_{FE}}{\partial\theta_{SP}} & \frac{\partial\tau_{FE}}{\partial\theta_{FE}} & \frac{\partial\tau_{FE}}{\partial\theta_{UR}}\\ \frac{\partial\tau_{UR}}{\partial\theta_{SP}} & \frac{\partial\tau_{UR}}{\partial\theta_{FE}} & \frac{\partial\tau_{UR}}{\partial\theta_{UR}}\end{bmatrix}\begin{bmatrix}\theta_{SP}\\ \theta_{FE}\\ \theta_{UR}\end{bmatrix} \tag{1}$$

Then, the following formula and table is obtained:

$$\begin{bmatrix}\tau_{SP}\\ \tau_{FE}\\ \tau_{UR}\end{bmatrix} = [A]\begin{bmatrix}\ddot{\alpha}\\ \ddot{\beta}\\ \ddot{\gamma}\end{bmatrix} + [B]\begin{bmatrix}\dot{\alpha}\\ \dot{\beta}\\ \dot{\gamma}\end{bmatrix} + [C]\begin{bmatrix}\alpha\\ \beta\\ \gamma\end{bmatrix} + [D] \tag{2}$$

[A] shows the inertial torque, [B] is the damping torque, [C] represents the stiffness torque, and [D] is for gravitational and torques associated with coupled rotations. The matrix values are given in the following equations. The inertial torque matrix values are

$$\begin{aligned} A_{11} &= 0.002\sin^2(\beta) + 0.00024\cos^2(\beta)\cos^2(\gamma) + 0.0016\cos^2(\beta)\sin^2(\gamma) + 0.0005\\ A_{11} &= 0.003\sin^2(\beta) + 0.0005\cos^2(\beta)\cos^2(\gamma) + 0.0027\cos^2(\beta)\sin^2(\gamma) + 0.0014 \end{aligned} \tag{3}$$

$$\begin{aligned} A_{12} = A_{21} &= 0.0014\cos(\beta)\cos(\gamma)\sin(\gamma)\\ A_{12} = A_{21} &= 0.0022\cos(\beta)\cos(\gamma)\sin(\gamma) \end{aligned} \tag{4}$$

$$\begin{aligned} A_{13} = A_{31} &= 0.002\sin(\beta)\\ A_{13} = A_{31} &= 0.003\sin(\beta) \end{aligned} \tag{5}$$

$$\begin{aligned} A_{22} &= 0.0016\cos^2(\gamma) + 0.00024\sin^2(\gamma) \\ A_{22} &= 0.003\cos^2(\gamma) + 0.0005\sin^2(\gamma) \end{aligned} \tag{6}$$

$$\begin{aligned} A_{23} &= A_{32} = 0 \\ A_{23} &= A_{32} = 0 \end{aligned} \tag{7}$$

$$\begin{aligned} A_{33} &= 0.002 \\ A_{33} &= 0.003 \end{aligned} \tag{8}$$

As stated above, the inertial torques for men and women are identical. The damping matrix is as follows (the first matrix represents the values for women and the second for men):

$$[B_{Women}] = \begin{bmatrix} 0.036 & 0.0038 & 0.006 \\ 0.0038 & 0.03 & -0.0035 \\ 0.006 & -0.0035 & 0.096 \end{bmatrix} \tag{9}$$

$$[B_{Men}] = \begin{bmatrix} 0.024 & 0.0008 & 0.008 \\ 0.0008 & 0.03 & -0.0032 \\ 0.008 & -0.0032 & 0.088 \end{bmatrix} \tag{10}$$

where the stiffness matrix is

$$[C_{Women}] = \begin{bmatrix} 0.827 & 0.08 & 0.15 \\ 0.08 & 0.71 & -0.08 \\ 0.15 & -0.08 & 2.24 \end{bmatrix} \tag{11}$$

$$[C_{Men}] = \begin{bmatrix} 0.756 & 0.017 & 0.29 \\ 0.017 & 0.99 & -0.099 \\ 0.29 & -0.099 & 2.24 \end{bmatrix} \tag{12}$$

and since the degrees of freedom are decoupled in this system, the D-matrix becomes as shown below by some simplifications, in which $K_{Women} = 0.17$ and $K_{Men} = 0.28$:

$$[D] = K \begin{bmatrix} \sin(\alpha)\sin(\gamma) - \cos(\alpha)\cos(\gamma)\sin(\beta) \\ \cos(\beta)\cos(\gamma)\sin(\alpha) \\ \sin(\alpha)\sin\gamma)\sin(\beta) - \cos(\alpha)\cos(\gamma) \end{bmatrix} \tag{13}$$

1.1. Control Architecture

The main architecture contains different subsystems. One part of that is calculating the resulting torque for the whole system. As shown in Figure S3, the hand model is updated in real time to calculate the required torque. The resulting torque can be calculated by subtracting the model-based output torque from the estimated muscle inserted torque.

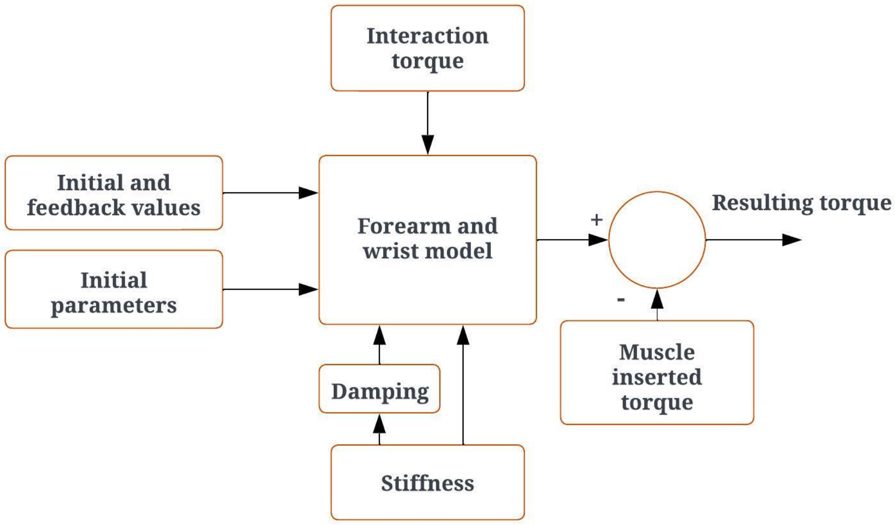

Figure S3. Resulting torque estimation.

2. Impedance Analyzer set up

The AD5933 impedance analyzer, used by PmodIA, has a 12-bit measurement range from 100Ω to 10MΩ and can excite an unknown impedance at a specific frequency. It uses SMA connectors for frequency input and response transmission, with the data processed using a discrete Fourier transform (DFT). We communicate with the analyzer via I2C interfaces, using the cost-effective Arduino Uno as the primary microcontroller. The schematic connection between PmodIA and Arduino is illustrated in Figure S4.

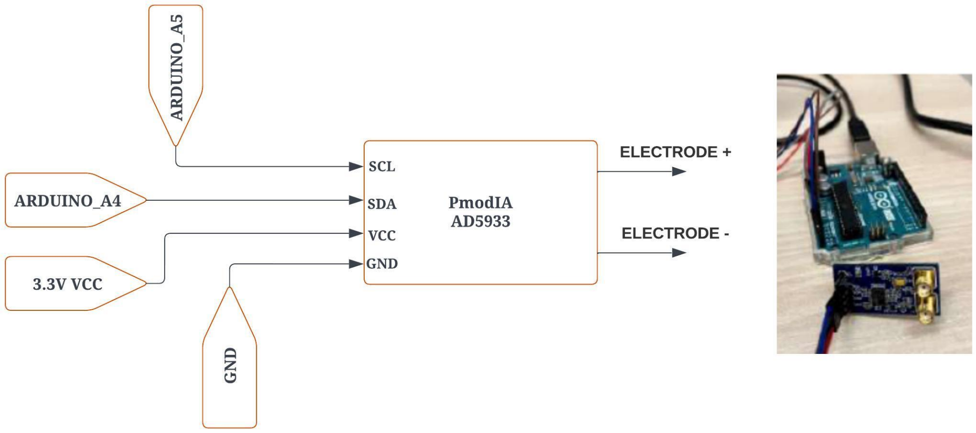

Figure S4. Connection of PmodIA AD5933 sensor and Arduino Uno.

3. Results and Discussion

To evaluate the controller's proficiency in adjusting force based on varying impedances, we examined it using two distinct impedance levels. It was posited that a system with elevated impedance should apply greater force to the hands, mirroring physiotherapist techniques. We established a force cut-off to ensure safety.

As shown in Figure S5, the resulting force is 16.98 N at 13 s. However, when the impedance increases, as seen in Figure S6, the resulting force is 21.24 N, an increase of 23%. Also, the torque is 1.36 at 15 s with lower impedance, but the torque is 1.58 Nm when the

impedance is higher. The force–torque sensor outcomes between the two impedance levels indicate that higher impedance correlates with increased force and torque.

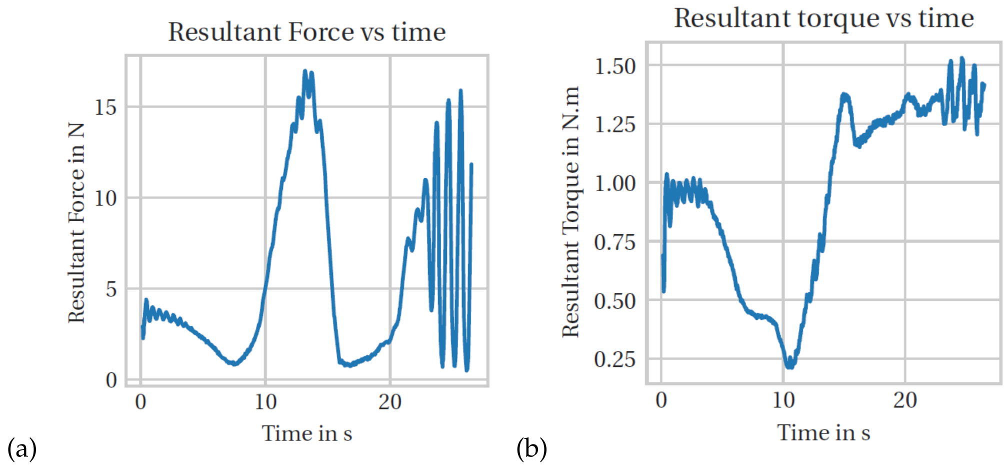

Figure S5. Corresponding forces and torque for lower impedance.

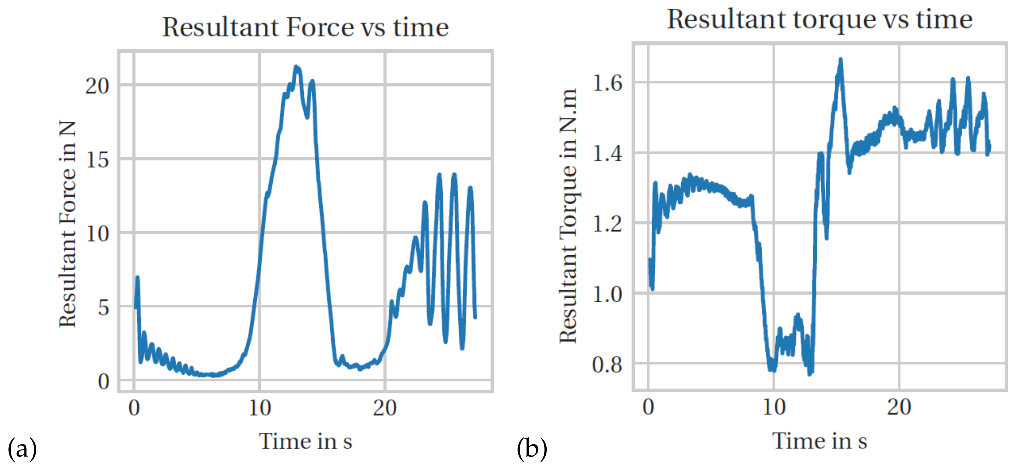

Figure S6. Corresponding forces and torque for higher impedance.
